# Deformation pattern in vibrating microtubule: Structural mechanics study based on an atomistic approach

**DOI:** 10.1038/s41598-017-04272-w

**Published:** 2017-06-26

**Authors:** Daniel Havelka, Marco A. Deriu, Michal Cifra, Ondřej Kučera

**Affiliations:** 1 Institute of Photonics and Electronics, The Czech Academy of Sciences, Prague, Czechia; 2 Istituto Dalle Molle di studi sull’Intelligenza Artificiale (IDSIA), Scuola universitaria professionale della Svizzera italiana (SUPSI), Università della Svizzera italiana (USI), Manno, Switzerland; 3 BIOCEV, Institute of Biotechnology, The Czech Academy of Sciences, Vestec, Czechia

## Abstract

The mechanical properties of microtubules are of great importance for understanding their biological function and for applications in artificial devices. Although microtubule mechanics has been extensively studied both theoretically and experimentally, the relation to its molecular structure is understood only partially. Here, we report on the structural analysis of microtubule vibration modes calculated by an atomistic approach. Molecular dynamics was applied to refine the atomic structure of a microtubule and a C_*α*_ elastic network model was analyzed for its normal modes. We mapped fluctuations and local deformations up to the level of individual aminoacid residues. The deformation is mode-shape dependent and principally different in α-tubulins and β-tubulins. Parts of the tubulin dimer sequence responding specifically to longitudinal and radial stress are identified. We show that substantial strain within a microtubule is located both in the regions of contact between adjacent dimers and in the body of tubulins. Our results provide supportive evidence for the generally accepted assumption that the mechanics of microtubules, including its anisotropy, is determined by the bonds between tubulins.

## Introduction

To cope with all of their functions in eukaryotic cells, microtubules (MTs) evince extraordinary mechanical characteristics^1^. They are very stiff compared to other components of the cytoskeleton, which helps them to bear high static stress^2^, yet they are also elastic enough to withstand dynamic loads. The properties of microtubules are length dependent^3, 4^, anisotropic^5^, and in contrast to their high stiffness, microtubules have been observed to be highly curved *in vivo* ^6^. Moreover, the mechanical characteristics of microtubules are tunable by either the binding of microtubule-associated proteins^7^ or possibly by post-translational modifications^6, 8^. The combination of these qualities enables microtubules to organize and maintain cell shape, to form the machinery responsible for chromosome separation during mitosis, to promote movement of flagella and cilia, and to constitute pathways for transport of cargo by motor proteins^9^. Many studies have been published aiming to explain the mechanical characteristics of microtubules observed in experiments^10^. The basics of microtubule mechanics are now fairly understood thanks to advanced mechanical engineering methods^11^. More elusive is how these properties are determined by the structural features of MTs on the molecular level.

In this study, we used atomistic modeling to investigate the deformation patterns of a microtubule obtained from the normal mode analysis of an entire microtubule. Previous theoretical works on microtubule mechanics have been focused either on global mechanical properties^11, 12^ or exclusively on the mechanics of individual inter-tubulin contact regions^13–17^; in contrast, this study was aimed towards a comprehensive description of strain in the molecular structure of microtubules. We have identified explicit regions of αβ-tubulin atomic structure that undergo both high compression and extension depending on the mode of motion of the microtubule. Since these regions are predominantly located at tubulin interfaces, it is evident that the mechanics of microtubules is dominated by bonds between tubulins instead of stiffness of tubulins themselves. This finding is of particular importance for (i) understanding the structural mechanics of microtubules on an intra-molecular level, and for (ii) prospective therapeutic targeting of microtubule mechanical properties, and for (iii) engineered modifications of microtubules in artificial applications.

## Results

**Figure 1:**
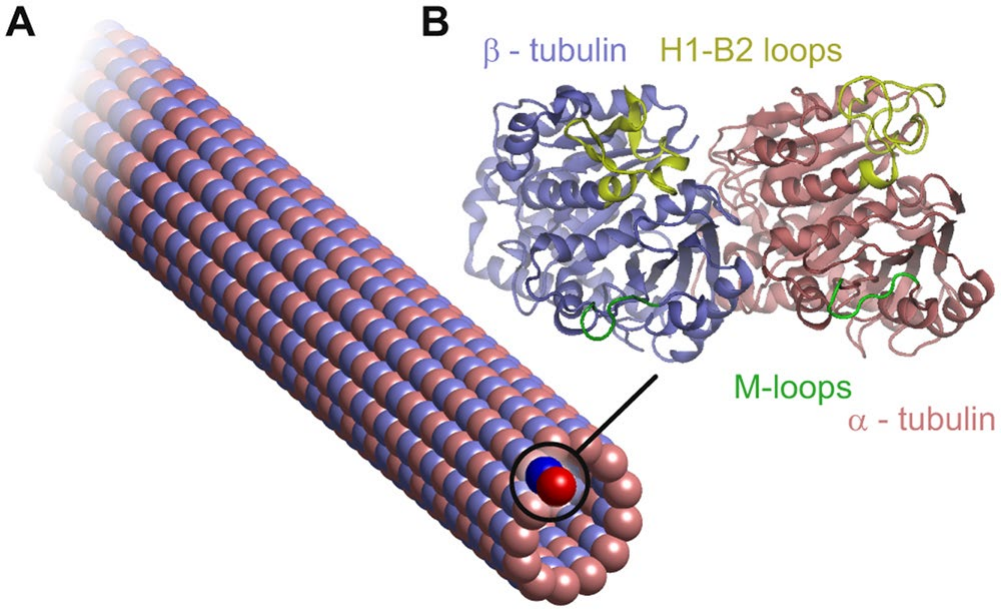
A schematic depiction of the structure of a microtubule with a 13:3 B lattice (**A**) and the secondary protein structure of an α-β tubulin dimer (**B**).

**Figure 2:**
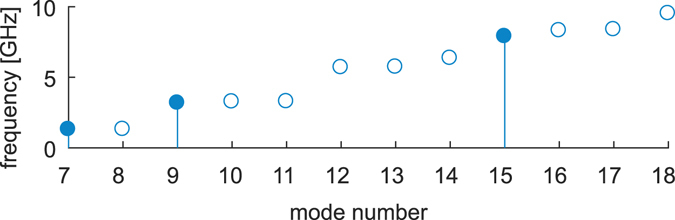
Frequencies of the first 12 nontrivial vibration modes of an approx. 320 nm long microtubule. The first nontrivial mode is mode number 7. The frequencies lie in a lower GHz range, which is in concordance with the predictions by other models. The three first modes of each type used in our analysis are depicted with line bars.

A numerical study using methodology described in detail bellow (see Methods) was performed on a microtubule containing 40 tubulin heterodimers in each protofilament and 520 dimers in total, where the total MT length was 320 nm (see Fig. 1). In accordance with previous studies, the vibration modes of microtubules of this length lie in the GHz range (see Fig. 2) and the mode shapes can be roughly divided into four groups: stretching, bending, torsional, and breathing modes. In contrast to continuum models, the mode shapes resulting from the elastic network approach have to be categorized visually. This issue, together with the dependency of the vibration frequency on the length of the microtubule, had been previously studied elsewhere^18^. It is important to emphasize that - due to the asymmetry of microtubules - some successive modes that could be considered degenerate on the basis of their global shape demonstrate subtle differences in their frequency and local shape.

**Figure 3:**
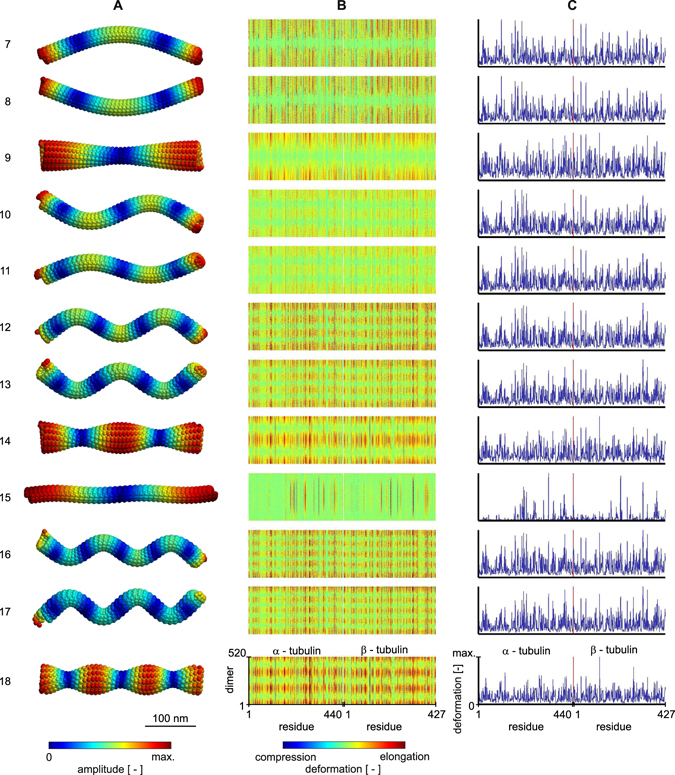
First twelve non-rigid normal modes of a microtubule. (**A**) the shapes of the modes with fluctuations mapped on the surface of the microtubule. (**B**) the deformation pattern in individual residues of αβ-tubulin dimers in the pseudo-helical lattice of a microtubule. Columns of the maps show residues within the sequence of tubulin and rows show individual dimers. (**C**) average absolute deformation in dimer. Red vertical line represents the boundary between tubulin monomers. Note that all deformation data are normalized to the maximum within respective mode.

In this study, we analyzed the first 12 non-rigid modes (mode numbers 7–18) shown in Fig. 3A. Three principal patterns of vibrations can be identified: an axial bending mode, a radial torsional mode, and an axial longitudinal stretching mode. This study has been focused on the detailed analysis of a single mode of each type. Specifically, we selected the principal bending (mode 7), torsional (mode 9), and stretching (mode 15) modes, which can all be identified within first 12 non-rigid modes in Fig. 3.

The calculated vibration modes show atomic group displacements within each mode; however, the fluctuation of a residue from its global equilibrium position does not provide a comprehensive view on the structural deformation of a microtubule undergoing vibrations. We therefore analyzed the displacement between each residue and its geometric nearest neighbors. Such an analysis effectively eliminates the translational and rotational movement of the rigid body on a local scale and is naturally related to the concept of elastic network models. The result of this procedure thus provides a good estimate of the deformation (or strain) of a microtubule lattice without extraordinary computational costs. The study of deformations of a microtubule using other protein-dynamics methods^19^ would require time-consuming computational operations (e.g. interpolation) over the dataset of eigenvectors representing the normal modes.

Deformations in the individual residues within all dimers are shown in Fig. 3B for modes 7 to 18. We follow gapless numbering convention of tubulin residues in the current paper, in contrast to Protein Data Bank entries 1JFF and JTUB which contain 2 gaps after β-Leu^44^ and 8 gaps after β-Pro^360^. The map of deformations clearly shows where the molecular structure of microtubule is compressed and where it is expanded. Absolute deformations averaged through all dimers (shown Fig. 3C) reveal the most stressed parts of the tubulin sequence. There is a huge variance in strain that residues experience, as some selected regions of each tubulin monomer sequence display high deformation while the rest of the sequence remains intact (see the vertical patterns in Fig. 3B). As seen in Fig. 3, the observed deformations are mode shape dependent and their absolute mean values are very similar within modes of the same type. Surprisingly, there are also distinct small parts of the tubulin sequence which share similar deformation response among different modes, as we show later.

**Figure 4:**
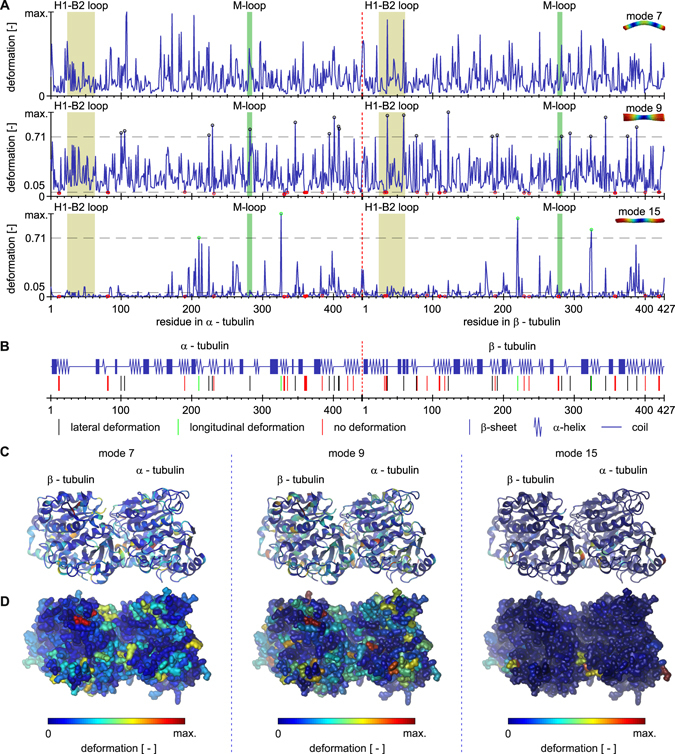
Average absolute value of deformation in tubulin dimers for three principal shapes of normal modes. (**A**) detailed view for the data shown in Fig. 3C. The average absolute deformation along tubulin dimer sequence is shown in blue. (**B**) specific regions of the sequence with substantial deformation are indicated in the secondary structure of tubulin dimer. They are also indicated with circles of respective color in (**A**), together with criterion thresholds. (**C**) average of absolute deformation is mapped on the ribbon model of tertiary structure of tubulin dimer and on tubulin surface model (**D**). Note that all deformation data are normalized to the maximum within respective mode.

In order to classify the regions relevant for deformations, we studied the three principal modes in detail: axial bending, radial torsion, and longitudinal stretching (see details of average absolute deformations in Fig. 4). A detailed look on the deformations in individual protofilaments in the case of bending mode (mode no. 7) reveals principal differences in the movement the residues undergo in respect to the direction of bending. At the moment of vibration shown in Fig. 4A, protofilaments in the plane of vibration following the direction of bending are compressed, whereas protofilaments on the opposite side are elongated. Protofilaments on the side of microtubule are deformed only marginally. In contrast to that, all of the protofilaments are uniformly elongated and have similar deformation in the stretching mode.

In the following, we classify deformations as longitudinal, lateral or negligible. The criterion for identification of residues undergoing lateral deformation in Fig. 4B is following: *i*-th residue manifests deformation magnitude, *d*_*i*_, such as ${d}_{i} > \,{\rm{\max }}\{d\}/\sqrt{2}$ only in mode 9. For longitudinal deformation, the criterion is: residue manifests deformation magnitude, *d*_*i*_, such as ${d}_{i} > \,{\rm{\max }}\{d\}/\sqrt{2}$ only in mode 15. For no deformation, the criterion is: residue manifests deformation magnitude, *d*_*i*_, such as ${d}_{i} < \,{\rm{\max }}\{d\}/20$ in both mode 9 and mode 15. There are no residues, which manifest both lateral and longitudinal deformation under given criteria. It is also observed that once residue manifests a lateral or longitudinal deformation, its longitudinal or lateral deformation, respectively, is negligible.

Under longitudinal stress (mode no. 15), the inter-tubulin longitudinal contact areas experience high deformation while there is only marginal deformation within the body of a tubulin dimer (see Fig. 4B and C). Compared to the longitudinal mode, the bending mode (mode no. 7) also causes deformation in the lateral inter-filament contacts, which is in concordance with the expected shear between filaments during bending^10, 20^. A comparison of the previous two modes with the torsional mode (mode no. 9) shows that the contact regions mentioned above are deformed. In contrast, however, the dominant deformation may be found in the inter-filament contacts while the longitudinal contacts experience weaker strain. The deformation in lateral contacts is discussed in detail in a later section.

Concerning the secondary structure of the tubulin, approximately one half of the deformations are localized in the α-helices, two fifths in the coils, and only one in ten takes place in the β-sheet. There is no substantial difference between α-tubulin and β-tubulin in this respect. Longitudinal deformations take place in very similar proportions regarding residues within coils, β-sheets and α-helices, with slight preference to the latter. In contrast to that, lateral deformations occur in residues predominantly within coils and α-helices. These different ratios are most likely caused by the fact that β-sheets form stiff core of the tubulin dimer which is unresponsive to shear between protofilaments. There is no general rule for the correspondence between the boundaries of deformed regions with the boundaries of the secondary structures, meaning that regions of high deformation pervade the sequence regardless of the form of the regular secondary structure.

Substantial deformations, i.e. those above $\mathrm{1/}\sqrt{2}$ of the maximum deformation within a mode, take place in only 26 residues out of 867 residues in tubulin dimer. From these, 4 residues deform exclusively under longitudinal loads and lateral deformations are unique to 22 residues. No residues undergo substantial deformations for both longitudinal and lateral stresses. There are also 34 residues with deformation lower than 0.05 of the maximum deformation within a mode, i.e. with nearly no deformation.

We analyzed deformations of selected amino-acid residues’ sequences that correspond to the regions of the tubulin important for a lateral mechanical connection of heterodimers in the microtubule lattice, i.e. between adjacent protofilaments. Deformations in these regions are of particular interest because of the recent controversy about the contribution of lateral contacts to the stability of microtubule lattice^21–23^. Lateral binding between tubulin heterodimers is mediated by M-loops, a sequence corresponding to residues 278–285 in α-tubulin and 276-283 in β-tubulin in our model, respectively. Another important region for lateral binding takes place in H1-B2 loop, residues α: 24–63 and β: 24–61. This loop has been shown to have a fluctuation maximum in the molecular dynamic study of tubulin sheets^18^. Both pairs of loops are depicted in Fig. 1A in green and yellow color, respectively. αTyr^282^ within the so called M-loop region deforms significantly under lateral stress. Although the H1-B2 region in the α-tubulin shows elevated deformation, it does not fall into $\mathrm{1/}\sqrt{2}$ criterion. A sequence αPro^359^-Val^362^, which is structurally located between lateral contacts in αM-loop and αH1-B2 loop, does not deform in response to longitudinal nor lateral stress, suggesting that absorption of deformation energy in lateral contacts prevent this sequence from deformation. The nucleotide binding site in αGly^142^-Gly^148^ shows lower deformation, however, it is slightly higher than the 0.05 criterion. Substantial deformation exists in β-tubulin in response to lateral stress in residues βTyr^36^ and βTyr^59^ in the so called H1-B2 loop and also in the residue βArg^282^ within the βM-loop. Residue βGly^277^ with no deformation can be also found in the βM-loop, demonstrating a huge diference in deformation even within one substructural element. Nucleotide binding site βGly^142^-Gly^148^ is again deformed only little, neither now fulfilling the 0.05 criterion.

**Figure 5:**
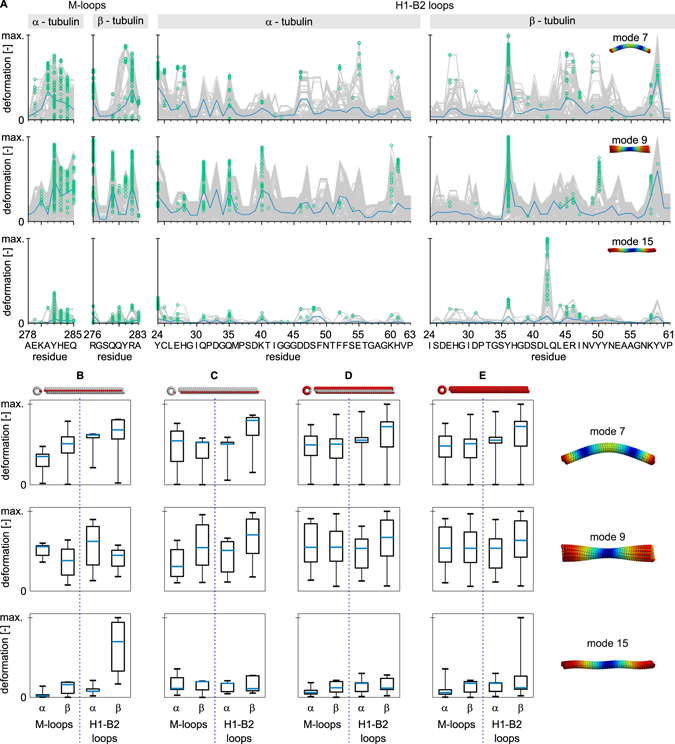
Deformation in lateral contacts for three principal shapes of normal modes. (**A**) absolute vibration-related deformations along residues forming the M-loop and H1-B2 loop within α and β tubulins. Absolute deformations for all tubulin dimers within microtubule (grey) with individual maxima used for box-plots bellow marked by green circles. The blue line shows the average of the absolute value of the deformation. Deformations in M-loops and H1-B2 loops in tubulins in the seam (**B,C**) and far from the seam (**D**) of the microtubule. Overall deformations in these regions are shown in column (**E**). The analyzed protofilaments are indicated in red above each column. The central blue mark in box-plots represents the median, the box represents a region within the 25th and 75th percentiles, and the whiskers represent the maximum extent of absolute deformation. Note that the deformations are normalized to the maximum within the respective vibration mode and that the number of tubulins within the seam is smaller than the number of tubulins not involved in the seam.

We were primarily interested in how the deformation in these regions differs for different mode shapes (see Fig. 5). The material experiences strain in different directions for different mode types, so we expected to observe slight differences in deformations in the M-loops compared to the H1-B2 loops due to the asymmetry of these regions within the microtubule lattice. Indeed, the deformation is slightly elevated in the H1-B2 loops in β-tubulins within the first axial bending mode (mode no. 7) compared to αH1-B2 loop. Since the bending of microtubules causes shear between protofilaments, the deformation in the M-loops is also pronounced. The torsional mode (mode no. 9) induces considerable deformation of all lateral contacts. Surprisingly, the longitudinal stretching mode (mode no. 15), which is not expected to show substantial shear between protofilaments, is connected with deformations in lateral contacts: however, the mean deformation is approx. three times smaller compared to bending and torsional modes (see Fig. 5E).

An important aspect of deformations in lateral contacts is the influence of positioning of a tubulin dimer within the microtubule B lattice. Tubulin dimers which put M-loops into a microtubule seam (Fig. 5C) do not show altered deformation pattern in these loops for bending and stretching deformations of the microtubule. However, the deformation in αM-loops is lower in these dimers for torsional stress. Dimers on the other side of the seam (Fig. 5B), i.e. those providing H1-B2 loops into the seam, show slightly altered deformations in M-loops in bending and torsional modes. Their response is also altered in αH1-B2 loops for torsional load and βH1-B2 loops for longitudinal load. This counter-intuitive result suggests that the forces balancing the discontinuity in the microtubule lattice propagate rather far in the microtubule structure.

To summarize, longitudinal stress causes strong few highly localized deformations in the inter-tubulin longitudinal contacts. Deformations responding to lateral stress are spread throughout the sequence with few maxima, some of which are localized in lateral contacts between tubulins in adjacent protofilaments.

## Discussion

The deformation in the tubulin sequence is mode-shape dependent and relates to a dominant direction of global mechanical stress. The deformations induced by normal modes of a microtubule tend to localize in the area of contacts between tubulin dimers. While stress in the axial direction results in deformations predominantly in longitudinal contacts, radial stress leads to deformation mainly in lateral inter-tubulin contacts. A substantial difference between the deformations in corresponding sequences in α and β tubulins within one mode is related to the dissimilar structure of these sequences and as a result, to different mechanical stiffness and different strength of the bonds to their surroundings. These results clearly illustrate how the inhomogeneity of a microtubule structure relates to inhomogeneous mechanical behavior.

The results described here cannot, by definition, be obtained from models based on mechanical continuum or coarse-grained discrete models. Such models are useful for the estimation of global movements; whereas the model applied here can predict motion of individual molecular residues, which is highly important for studying the functional relevance of such motions. The price paid for this accuracy is the computational power needed for thorough analysis. Nevertheless, the increasing availability of high performance computing facilities makes this approach ordinarily applicable.

The mechanical properties of a microtubule are not only determined by mutual interactions of tubulin dimers (given, besides other things, by geometry of microtubules), but also by their mechanical characteristics. From this point of view, it is interesting to compare dynamics of a polymerized microtubule with dynamics of tubulin dimer, which has been studied both by molecular dynamics simulations^24^ and normal mode analysis^25^. These studies have shown that the regions of tubulin sequence with the highest mobility are mainly those involved in contact with adjacent dimers in polymerized microtubule. This is in good accordance with our results, which show the high deformations in contact regions. In contrary, fluctuations of individual residues in free and bounded dimer are completely different because they are given by global shape of normal modes.

Since microtubules are subject to both static and dynamic loads, their vibration characteristics are of great importance. Due to obvious experimental difficulties, the vibration characteristics of microtubules have been only studied theoretically until recently^11, 26^. There are several approaches to modeling the natural vibrations of microtubules. Continuous models approximate microtubules by a mechanical continuum in the form of beams or shells^27, 28^. During the past two decades, these models have been advanced to include all of the important properties of microtubules, including anisotropy, nonlinearity, and the effects of surroundings. Examples of these modes encompass Euler-Bernoulli^29–33^, Timoshenko beam models^29, 33–36^, orthotropic shell models^29, 33, 37–48^. The main advantage of continuous models resides in their relative efficiency and in some cases, the existence of an explicit analytic solution. Even so, these models have a limited ability to predict the structural motions on the level of molecular groups within microtubules.

Discrete models of microtubule mechanics have evolved from the initial chain-of-particles model^49^, through more detailed models^50^, to a fully atomistic description of the microtubule normal modes^18^; models that provide high resolution at the cost of requiring high computational power. As a compromise between these two approaches, hybrid methods based on an atomistic-continuum approach have been developed to model the vibration characteristics of microtubules^51–54^. Nevertheless, a high resolution vibration analysis by any of these aforementioned methods and mapped to the level of individual molecular groups within heterodimers has not been published until now.

Our methods and results expand on work by Deriu *et al*.^18^. The main contribution of present work is the systematic analysis of the deformation within a microtubule. The major improvement of the methods used herein is that the tubulin C-terminal tails were considered in molecular dynamics, which was performed for 25 ns instead of 10 ns. All atom trajectories were reconstructed from C_α_ trajectories.

## Conclusion

Here, we reported a high resolution analysis of the normal modes of a microtubule calculated by an atomistic approach, with special attention to the deformation of the microtubule body. With the aid of molecular dynamics, we built an atomic precision model of a microtubule and performed a normal mode analysis of the resulting structure. Our results show a detailed structure of the lowest vibration modes having a resolution of molecular groups within individual heterodimers. The knowledge of the atomic groups’ displacements within vibration modes is particularly important for the validation of other computational approaches and furthermore, for the assessment of the biological relevance of microtubule vibrations through structural motions on the level of amino-acid residues. It is assumed that the vibration modes of proteins have functional relevance in biology^55–58^ and it seems reasonable that the vibrations of protein-composed structures have this relevance as well. In the deformation analysis reported here, we identified well defined regions of αβ-tubulin atomic structure that undergo both high compression and extension depending on the mode of motion of the microtubule. Since these regions are predominantly located at the surface area of tubulins in the contact regions, it is evident that inter-tubulin bonds are softer than the tubulin itself. Surprisingly, our results indicate that the deformation is substantially different in α-tubulins compared with β-tubulins.

The study of the structural mechanics of microtubules is important for the comprehension of their versatile biological functionality. In this context, understanding the deformation pattern of a microtubule on a molecular basis is relevant to prospective targeting of microtubules in medical therapeutic strategies or for their use in nanoengineering. This study has shown for the first time the structural deformation analysis of microtubule vibration modes calculated by an atomistic approach. The main advantage of the method described here resides in the fact that it (i) does not require *a priori* knowledge about the mechanical parameters of the microtubule and that (ii) it enables the study of internal mechanical deformation within individual tubulin monomers. The results presented here localize mechanical strain within the molecular structure of a microtubule to interdimer contacts, which presents evidence that the anisotropic mechanics of microtubules result from contacts between dimers.

Research in the field of microtubule vibrations allows for better understanding of microtubule mechanics, which has fundamental importance in cell biology and in microtubule-inspired or microtubule-targeted engineering. Future research of microtubule vibrations should bring experimentally testable predictions regarding biological relevance of microtubule vibrations beyond global material properties, as the research on the level of individual proteins indicates. If there is any functional significance of microtubule vibrations themselves, it should be, in concordance with the molecular biology paradigm, researched on the level of individual molecular groups.

## Methods

The basis for the development of an atomistic model of a microtubule was the selection and refinement of the α-β tubulin dimer. The αβ-tubulin atomic structure was obtained from the Protein Data Bank (crystal structure of bovine brain tubulin 1JFF^59^). The tubulin structure was repaired via basic homology modeling by adding the missing residues from 1TUB^60^ following a procedure already employed in previous literature^18, 61^. In addition, tubulin C-terminal tails were added starting from the primary sequence contained in 1JFF.

Next, we mapped the tubulin dimer atomic structure into the structure of a whole microtubule. Repaired α- and β- tubulin monomers were docked onto a 13:3 B lattice microtubule model^62^ following the same approach reported in previous work^18^. In detail, each α/β tubulin monomer was superimposed to all corresponding monomers in the whole microtubule structure by using the GROMACS tool g_confrms, by computing the root mean square deviation (RMSD) of two structures after a least-squares fitting of the second structure (monomer taken from the repaired 1JFF) on the first one (the same monomer on the whole microtubule structure). The group for the least-square fitting is defined as the C_α_ atoms of the β-sheets on the monomer central core.

**Figure 6:**
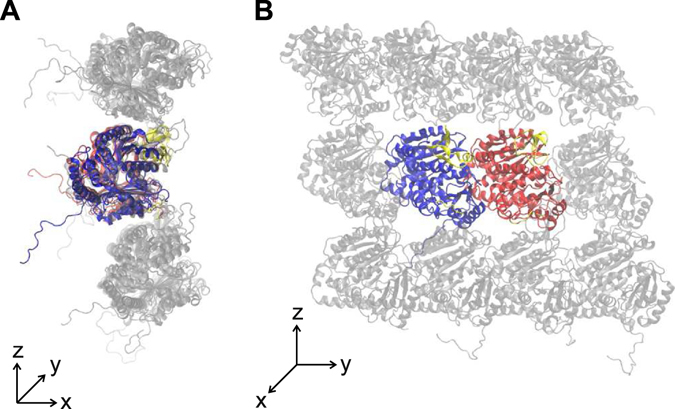
An example of the simulated tubulin system composed of 12 monomers (the S1 system is shown). The surrounding monomers are shown in gray. Tubulin tails are modeled and free to fluctuate and rearrange during the simulation. The system is shown in the plane perpendicular to the microtubule axis (**A**) and parallel with the axis (**B**).

From this whole microtubule atomic structure we extracted 3 systems composed of 12 monomers as shown. Each system consists of a central tubulin dimer completely surrounded by adjacent monomers, in both the longitudinal and circumferential directions following the curvature of a 13:3 B microtubule. Hence, the 3 systems are built of 12 interacting monomers as the microtubule wall: (S1) far from the microtubule seam (see Fig. 6); (S2) within the microtubule seam with the central dimer experiencing αα/ββ contacts on one side and αβ/βα contacts on the other side; and (S3) within the microtubule seam with the central dimer experiencing αβ/βα contacts on one side and αα/ββ contacts on the other side. The above listed 3 configurations represent all possible interaction configurations for a dimer when present in the 13:3 B microtubule lattice.

Each system (S1, S2, S3) was fully solvated in explicitly modeled water^63^ where the minimum distance between the protein complexes (12 monomers) and the edge of the box was at least 1 nm. The net charge of the system was neutralized by the addition of Cl^−^ and Na^+^ ions. The above mentioned systems (each consisting of about 360,000 interacting particles) were first minimized by the steepest descent energy minimization algorithm followed by a preliminary position restraint molecular dynamics (MD) simulations of 100 ps (force constant equal to 1,000 kJ/(mol · nm^2^)). Then, a short MD of 1 ns duration without restraints was run in the NpT, or isothermal-isobaric, ensemble. Finally, a production run was performed for 25 ns in the NVT, or canonical, ensemble. During the production MD, restraints (force constant equal to 100 kJ/(mol · nm^2^)) were still applied to C_α_ atoms of beta sheets pertaining to the monomers’ central core. The aim of the production molecular dynamics was only to let the protein rearrange the aminoacids involved in lateral and longitudinal tubulin-tubulin interaction. The GROMACS 4.6 package has been used for all MD simulations and data analysis^64^. Gromos54a7 FF had been employed to prepare the protein topology. GTP and GDP force fields were obtained by using the Automated force field Topology Builder web server^65^. Electrostatic interactions were calculated at every step with the Particle-Mesh Ewald method with a short-range electrostatic interaction cut off of 1 nm. A cut-off of 1 nm was also applied to Lennard-Jones interactions. By associating heavy atoms and a virtual site approach^66^ to the LINCS constraint solver (selecting all-bonds constraints)^67^, a time step of 4 fs was used. The GROMACS *v-rescale* thermostat^68^ was used to keep simulation temperature constant at 300 K. The tubulin monomer relative position is supposed to be achieved by mapping each tubulin monomer on the entire microtubule structure.

The central dimer of each system was mapped to the corresponding dimer in the microtubule structure as done in the very beginning. The microtubule part far from the seam was reconstructed by a central dimer of the S1 system, while dimer pertaining to protofilaments on the seam are reconstructed by mapping the central dimer of system S2 and S3.

A normal mode analysis of a whole microtubule had been carried out by following the elastic network approach, which requires a simplification of the structure from an all-atom model to a C_α_ model, with removed flexible C-terminal tails. The C_α_ atoms within a cutoff distance of 1.2 nm were coupled by linear springs with force constant $\gamma$, which corresponds to quadratic potential with elastic constant equal to 1 kcal/(mol $\cdot$ Å^2^). The potential energy, ${V}_{p}$, of such a system may be written as

1 $${V}_{p}=\frac{1}{2}{\rm{\Delta }}{{\bf{r}}}^{T}{\bf{H}}{\rm{\Delta }}{\bf{r}}$$

where ${\rm{\Delta }}{\bf{r}}$ is a fluctuation vector of C_α_ atoms and ${\bf{H}}$ is a Hessian matrix comprised of second derivatives and second mixed derivatives of the potential energy with respect to coordinates of ${\bf{r}}$ ^69^. Eigendecomposition of ${\gamma }^{-1}{\bf{H}}$ yields set of eigenvalues ${\lambda }_{i}$ corresponding to vibration frequencies ${\omega }_{i}=\sqrt{\gamma {\lambda }_{i}}$ and eigenvectors ${{\bf{e}}}_{i}$ of ${\gamma }^{-1}{\bf{H}}$ reflecting shapes of vibration modes.

An approximation of the all-atom trajectory from a C_α_ trajectory was obtained by reconstructing the all-atom structure for each tubulin dimer of the whole microtubule starting from all-atom information by means of TM-align^70^. This had been done for each mode after decomposition.

Standard flexibility analysis requires interpolation of the mode eigenvectors into a regular grid. It is rather impractical in our case because the eigenvectors have size of $N=\mathrm{450,840}$. By definition, deformation is the difference in distance between two adjacent particles of the body. In direct connection with the elastic network concept, we estimated deformations, *D*, in a microtubule by averaging displacements of the *k*-th amino acid residue with position vector ${{\bf{r}}}_{k}$ from its geometrically nearest neighbor with position vector ${{\bf{r}}}_{ki}$ as

2 $${D}_{k}=\Vert {{\bf{r}}}_{k}^{^{\prime} }-{{\bf{r}}}_{ki}^{^{\prime} }\Vert -\Vert {{\bf{r}}}_{k}-{{\bf{r}}}_{ki}\Vert $$

where the apostrophe indicates coordinates within the deformed state.

We used VMD for visualization of the structure of tubulin^71^ and STRIDE for secondary structure prediction^72^. All data analysis was performed off-line using a commercial software package (MATLAB Release 2015a, The MathWorks Inc., Natick, MA, USA, 2015). MAVEN package^73^ was used for structural data mapping.
